# MrSVP, a secreted virulence-associated protein, contributes to thermotolerance and virulence of the entomopathogenic fungus *Metarhizium robertsii*

**DOI:** 10.1186/s12866-019-1396-8

**Published:** 2019-01-28

**Authors:** Tian Xie, Yulong Wang, Deshui Yu, Qilin Zhang, Tingting Zhang, Zhangxun Wang, Bo Huang

**Affiliations:** 1 Anhui Provincial Key Laboratory of Microbial Pest Control, Anhui Agricultural University, Hefei, 230036 China; 2 School of Plant Protection, Anhui Agricultural University, Hefei, 230036 China

**Keywords:** *Metarhizium robertsii*, Secreted protein, Thermotolerance, Virulence

## Abstract

**Background:**

*Metarhizium robertsii*, a widely distributed insect pathogen, is presently used as a natural alternative to chemical insecticides. Unfortunately, its worldwide commercial use has been restricted by a short shelf life and inconsistencies in virulence. In our previous study, a gene (GenBank accession number EFZ01626) was found to be significantly upregulated in heat-treated conidia. In the present study, this gene was characterized via gene disruption and complementation strategies.

**Results:**

The gene (amplified by rapid amplification of cDNA ends PCR) was 1219 bp long and contained an open reading frame (ORF) of 777 bp. It encoded a protein of 234 amino acid residues with a 26-residue signal peptide. Bioinformatics analyses did not identify conserved functional domains; therefore, it was assumed to be a secreted virulence-associated protein according to its signal peptide and bioassay results. We found that the conidial germination rate of the *ΔMrSVP* mutant fungi dramatically decreased after heat shock treatment in a thermotolerance test. In addition, transcription levels of all tested heat shock–related genes were significantly lower in the mutant than in the wild type. We also demonstrated that the mean lethal time to death (LT_50_) of *ΔMrSVP* significantly increased relative to the wild type in insect bioassays (both topical inoculation and injection) involving *Galleria mellonella*. Moreover, similar rates of appressorium formation between *ΔMrSVP* and the wild type—and the significantly different expression of virulence-related genes such as acid trehalase and sucrose nonfermenting protein kinase in the haemocoel after injection—revealed that MrSVP is required for virulence in the insect haemocoel.

**Conclusions:**

Overall, our data suggest that the *Mrsvp* gene contributes to thermotolerance and virulence of *M. robertsii*. Furthermore, this gene is deeply involved in the mycosis of insect cadavers and in immune escape rather than insect cuticle penetration during infection.

**Electronic supplementary material:**

The online version of this article (10.1186/s12866-019-1396-8) contains supplementary material, which is available to authorized users.

## Background

*Metarhizium robertsii*, the model species of an entomopathogenic fungus, has many advantages, such as a wide host range, environment-friendliness, safety to humans and other animals, and easy production and preparation. Unfortunately, commercialized worldwide use of fungal biocontrol agents is partially limited by the failure of conidia to retain viability during long-term storage, transportation, and use under high-temperature conditions [1, 2]. Moreover, the relatively unstable virulence of *M. robertsii—*as a fungal agent that uses living cells as the insecticidal component [3]—is also cited as a critical obstacle to commercialization of this organism. Therefore, understanding the biological function of *M. robertsii* genes involved in thermotolerance and virulence will help to improve preparation quality and stability of insecticidal efficacy of this fungal agent.

Genomic sequencing has revealed a total of 11,689 proteins in the *M. robertsii* genome, including 1278 secreted proteins, and a strikingly larger proportion of genes encoding secreted proteins relative to other fungi [4]. Many of the secreted proteins belong to families that may have roles in colonization of insect tissues, e.g. proteases [5]. Infection of an insect by *M. robertsii* is mainly subdivided into three steps: insect cuticle penetration; mass-propagation and secretion of toxins in the insect body; penetration of the cuticle again, and production of spores. To date, proteins secreted during the insect cuticle penetration stage, e.g., protease, chitinase, lipase, esterase, and other cuticle-degrading enzymes, have been widely studied [6]. Lately, there is also some progress on elucidation of the *Metarhizium* adhesion process [7] via which *M. anisopliae* adheres to the insect epidermis or plant surface depending on two hydrophobic proteins, MAD1 and MAD2 [8]. Following penetration, *Metarhizium* propagates in the nutrient-rich insect haemocoel via immune evasion and adaptation to osmotic stress [9], which is also involved in expression of the secreted proteins. First, *Metarhizium* can secrete a protein similar to host collagen (MCL1) onto the surface of its cell to avoid recognition and encapsulation by insect immune cells [10]. Then, the species can express the MOS1 receptor protein to adapt to the high osmotic pressure of hemolymph. After that, it can grow by means of secreted acid phosphatase and trehalase to degrade organic phosphorus and trehalose, respectively, in the haemocoel of insects [11]. At last, the fungi eventually secrete a large number of secondary metabolites to interfere with, inhibit, or counter the host immune response and to kill the insect.

In this study, a gene (EFZ01626) with upregulated expression (more than 1000-fold) in heat-treated conidia of *M. robertsii* was found by high-throughput RNA sequencing (RNA-Seq) [12] and then identified as a gene with secreted-protein structure (named as *Mrsvp*) in *M. robertsii*. Here, the functions of *M. robertsii Mrsvp* were analyzed via a gene knockout: tolerance of the mutant strain to heat and the ability to kill *Galleria mellonella* were significantly weaker.

## Results

### Identification of the *Mrsvp* gene and sequence analysis

The sequencing results on the 3′- and 5′-RACE-amplified fragments revealed that the cDNA sequence is 1219 bp long (GenBank accession number EFZ01626). The open reading frame consists of 777 nucleotides, and the 5′ untranslated region (UTR) and 3′-UTR are 278 and 164 bp, respectively. Downstream of the termination codons, the 3′-UTR contains a polyA tail signal sequence (ACTAAA) and a 20 bp polyA structure. The gene encodes a protein of 234 aa with a calculated MW of 23.931 kD and its isoelectric point is 6.698. Bioinformatics analyses revealed that there is a signal peptide sequence (from aa position 18 to 43), and conserved functional domains were not identified. According to the experimental results that the gene affects virulence [13], it was assumed to be a secreted virulence-associated protein and named as MrSVP.

### Construction and validation of the *Mrsvp* knockout and complementation strains

To characterize the functions of MrSVP in *M. robertsii*, the gene replacement mutant was obtained by means of a dominant selectable marker gene, *bar*. The Δ*MrSVP* deletion mutants were constructed by replacement of the coding *Mrsvp* gene regions with the *bar* gene cassette. Complementation strains (Δ*MrSVP*/ *MrSVP*) were obtained by transfecting the pben-SVP vector into the Δ*MrSVP* mutant.

All the recombinant strains were confirmed by PCR and RT-PCR. PCR analysis indicated that a 692 bp fragment corresponding to the partial *Mrsvp* gene was present in the WT and Δ*MrSVP*/*MrSVP* but not in strain Δ*MrSVP* (Additional file 1: Figure S1B and C). A *bar* gene fragment (410 bp) is present in strains Δ*MrSVP*, Δ*MrSVP*/*MrSVP*, and a *ben* gene fragment (328 bp) is present only in strain Δ*MrSVP*/*MrSVP* (Additional file 1: Figure S1B and C).

### *Mrsvp* contributes to thermotolerance but is not involved in UV stress

**Fig. 1:**
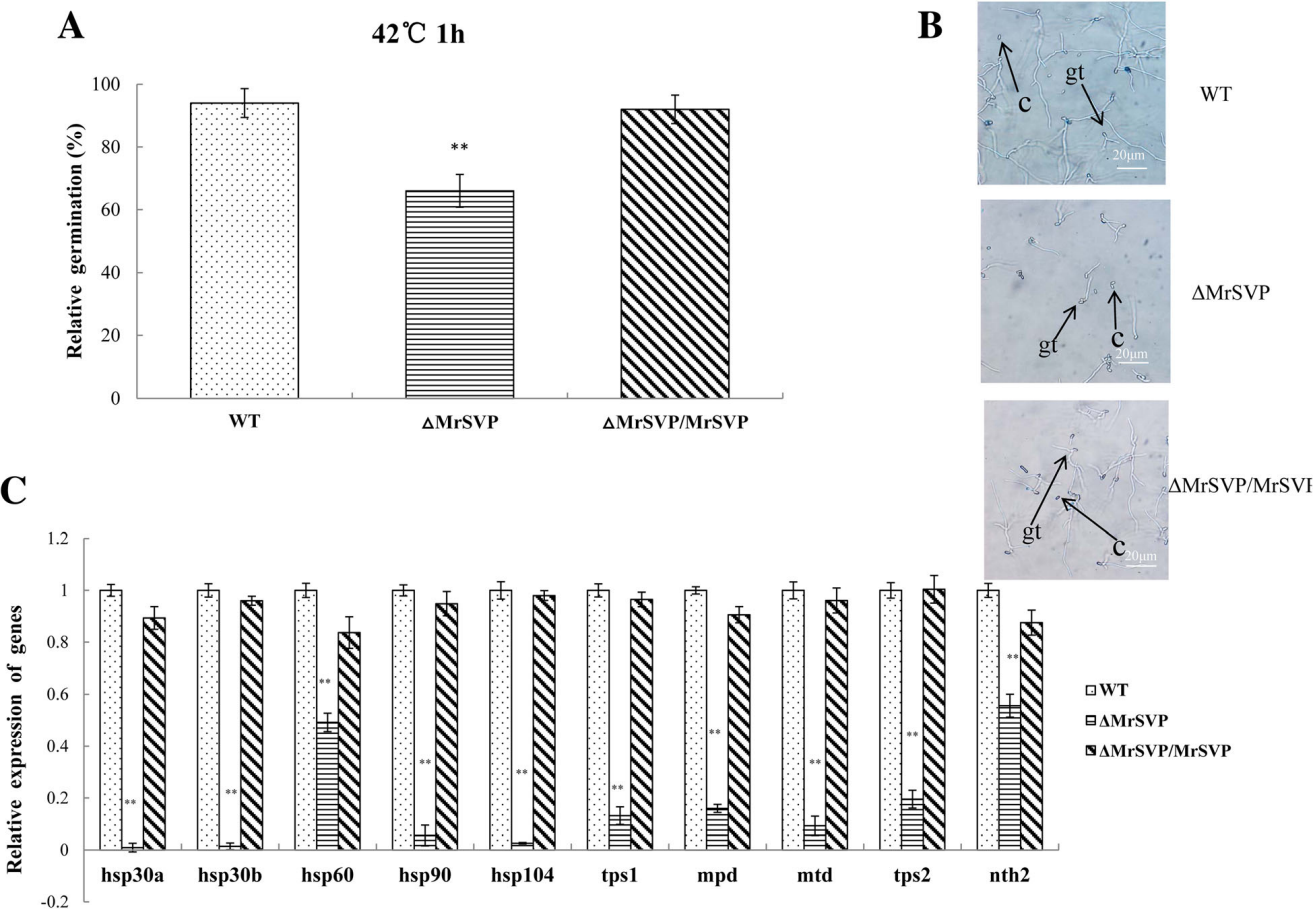
The relative percentage of germination of *M. robertsii* conidia after heat shock treatment. **a** The mean relative percentage of germination of *M. robertsii* conidia after exposure to heat shock. Based on controls that were not treated with heat shock, relative germination was calculated. Error bars: SD of the mean from three replicate assays. **b** The germination of conidia was observed by microscopic examination after heat shock, gt: germ-tube; c: conidia. **c** Expression analysis of the genes related to tolerance to heat shock. After 60 min at 42 °C, and 24 h incubation at 25 °C, mRNA transcripts were quantified by real-time RT-PCR

Loss of *Mrsvp* resulted in a decrease of conidial thermotolerance at 42 °C; 66.15% (*P* < 0.01) of the Δ*MrSVP* conidia germinated relative to freshly prepared control, whereas 94.28 and 92.33% of the WT and Δ*MrSVP*/ *MrSVP* conidia germinated relative to freshly made control, respectively (Fig. 1a).

The expression of genes related to tolerance to heat shock in the fungus was detected by real-time RT-PCR [14]. The relative expression levels of five heat shock proteins and five genes related to heat tolerance in mutant Δ*MrSVP* were significantly lower than those in WT and Δ*MrSVP*/*MrSVP* (*P* < 0.01; Fig. 1c). This finding was consistent with a decrease in conidial thermotolerance in the mutant. These data indicated that *Mrsvp* plays an important part in conidial tolerance to thermal stress.

The tolerance of conidia to UV irradiation among strains WT, Δ*MrSVP*, and Δ*MrSVP*/*MrSVP* was not obviously different. The relative germination rates of conidia were approximately 79, 69, and 80% for strains WT, Δ*MrSVP*, and Δ*MrSVP*/*MrSVP*, respectively, when the conidia were treated with UV-B irradiation (Additional file 1: Figure S3B). Further analysis of variance of these data showed that there is no significant difference in the tolerance to UV among our strains: the mutant, WT, and complementation strain.

### *Mrsvp* is required for virulence in the insect haemocoel

**Fig. 2:**
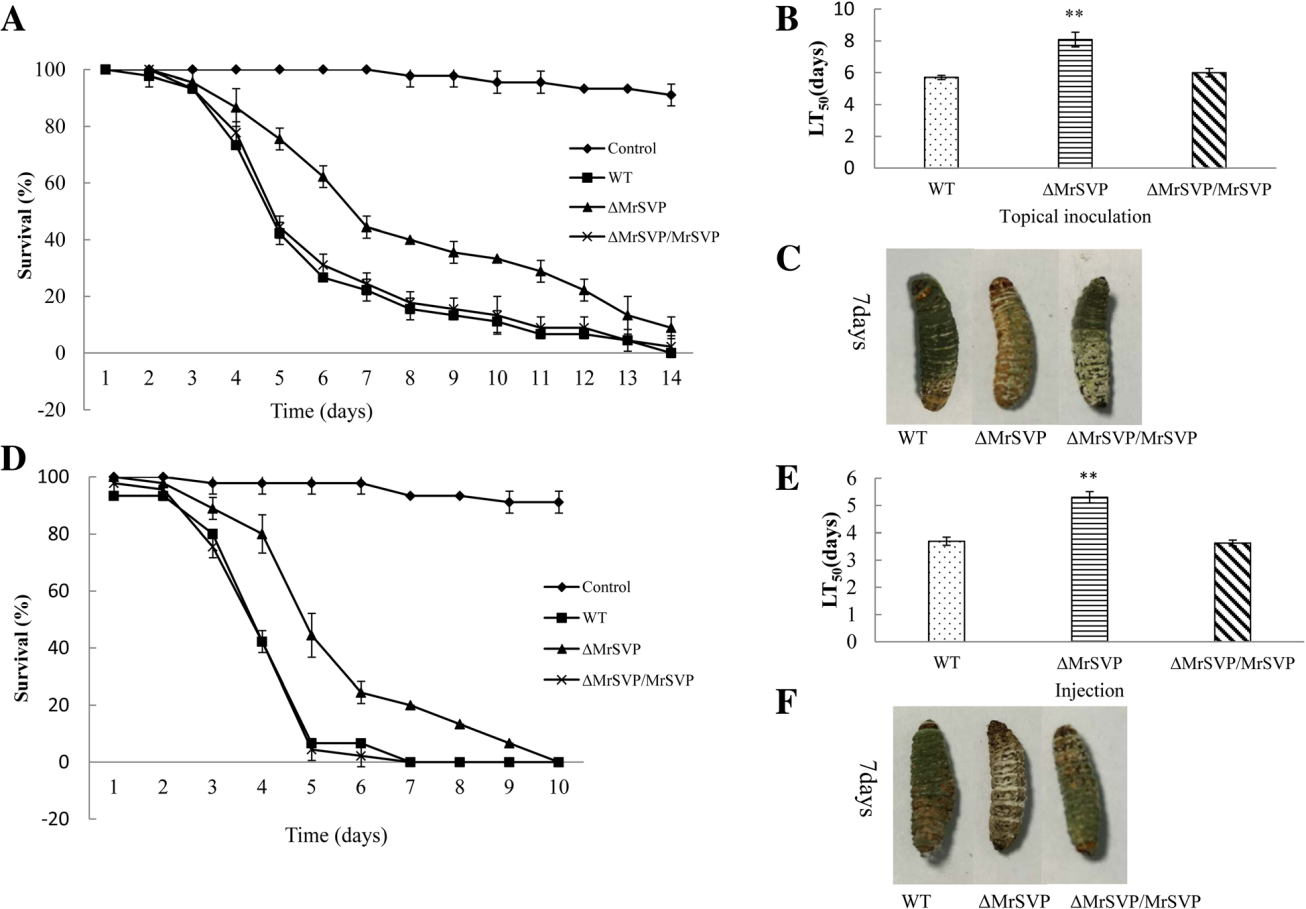
Insect bioassays. **a** Survival of *G. mellonella* after topical inoculation of conidia of WT, Δ*MrSVP*, or Δ*MrSVP*/*MrSVP*. *G. mellonella* specimens were treated for 60 s with sterile distilled H_2_O as a control. **b** LT_50_ for topical inoculation assay. **c** Representative images of the host cadavers treated with the indicated strains. **d** Survival of *G. mellonella* after injection of conidia of WT, Δ*MrSVP*, or Δ*MrSVP*/*MrSVP*. *G. mellonella* specimens were injected with 10 μl of sterile distilled H_2_O as a control. **e** LT_50_ for the injection assay. **f** Representative images of the host cadavers treated with the indicated strains. Error bars indicate standard errors of three trials. Capital letters on bars denote significant differences between samples (*P* < 0.01)

Mutant Δ*MrSVP* manifested severely reduced virulence as compared with the WT in infection bioassays based on the greater wax moth, *G. mellonella*. For topical inoculation, comparisons of LT_50_ values showed that Δ*MrSVP* (LT_50_ = 8.08 ± 0.46 d) took significantly longer (41.75%) to kill as compared with the WT (LT_50_ = 5.70 ± 0.12 d) (*P* < 0.01; Fig. 2a). Regarding the injection, the differences were also significant (43.24%) between the WT (LT_50_ = 3.70 ± 0.13 d) and Δ*MrSVP* (LT_50_ = 5.30 ± 0.20 d; *P* < 0.01; Fig. 2d).

**Fig. 3:**
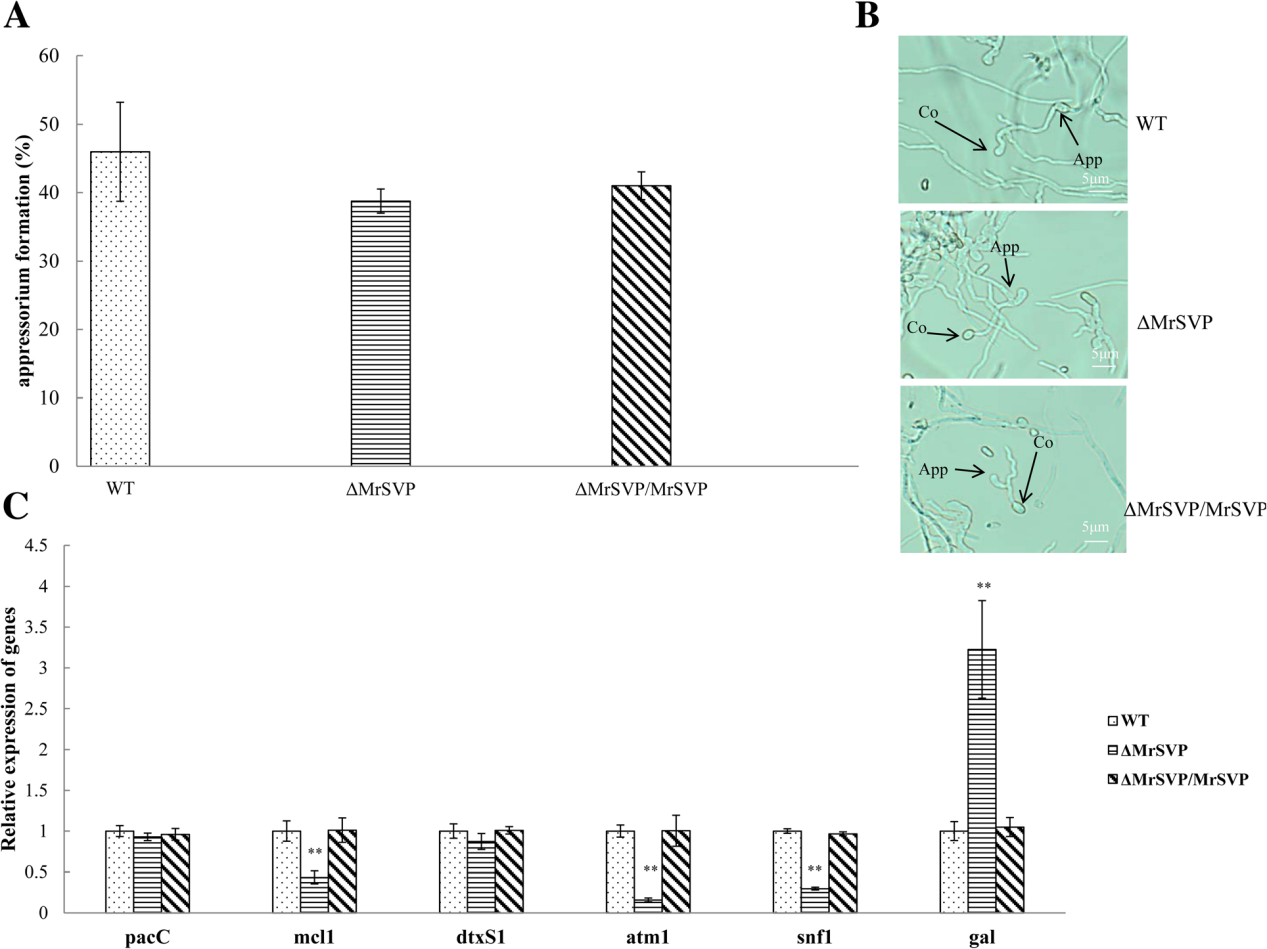
*Mrsvp* disruption affects the fungal virulence in insect hemolymph. **a** The appressorium formation rate at 36 h after treatment. **b** Appressorium development was observed by microscopic examination after treatment. App, appressorium; Co, conidium. **c** Expression analysis of the virulence-related genes in the haemocoel. The relative expression levels of the *gal* gene were employed to quantify the transcripts of *GAL* in the strains WT, Δ*MrSVP*, and Δ*MrSVP*/*MrSVP*. mRNA transcripts were quantified at 48 h after injection by real-time RT-PCR

The expression levels of genes related to virulence of *M. robertsii* in the insect haemocoel were detected by real-time RT-PCR. The relative expression levels of the genes involved in nutrition utilization or immune escape were significantly lower in mutant Δ*MrSVP* than in the WT and Δ*MrSVP*/*MrSVP*. Moreover, the expression level of the antifungal peptide of the insect was significantly increased (*P* < 0.01; Fig. 3c). In addition, with respect to appressorium formation, 44.17% of the germinated conidia from Δ*MrSVP* formed typical appressoria, and there was no significant difference from the WT (52.78%) and Δ*MrSVP*/*MrSVP* (50.34%, *P* > 0.05).

These experimental data confirmed that the *Mrsvp* gene contributes to virulence of *M. robertsii*; furthermore, the gene is deeply involved in mycosis of insect cadavers and in immune escape rather than insect cuticle penetration during infection.

### Analyses of growth rates, sporulation, and chemical stress

In PPDA, PDA, SDAY, and 1/4 SDAY (four culture media), the WT and knockout strain (Δ*MrSVP*) and complementation strain (Δ*MrSVP*/*MrSVP*) grew normally.

After examination of the morphology of colonies, of the density and height of aerial hyphae, of the shape of a colony edge, and the color of colonies, no obvious differences were found. By means of the medium diameter measurement in the mutant strain, and using the complemented strain and WT strain in four different culture media, we found that the colony diameters of the three strains were similar (Additional file 1: Figure S2A). When conidia from the mutant Δ*MrSVP*, Δ*MrSVP*/*MrSVP*, and from the WT were quantified compared with the yield obtained from the WT on SDAY, 1/4 SDAY, PPDA, and PDA media, the conidial yields of mutant Δ*MrSVP* were lower by 8.34, 18.54, 18.56, and 9.89%, respectively (Additional file 1: Figure S2B). There was no significant difference in variance analysis. Therefore, gene *Mrsvp* is not involved in vegetative growth and sporulation of *M. robertsii*.

Effects of osmotic, oxidative, cell wall, antioxidant capability, and fungicidal stressors were examined on a PDA containing NaCl, H_2_O_2_, SDS, and Congo Red, menadione, and carbendazim respectively, with the data presented as radial growth (colony diameter). Contrary to our expectations, the colony diameter of the Δ*MrSVP* mutant turned out to be similar to that of the WT and Δ*MrSVP*/*MrSVP* in the presence of stressful chemicals (Additional file 1: Figure S3A).

## Discussion

**Fig. 4:**
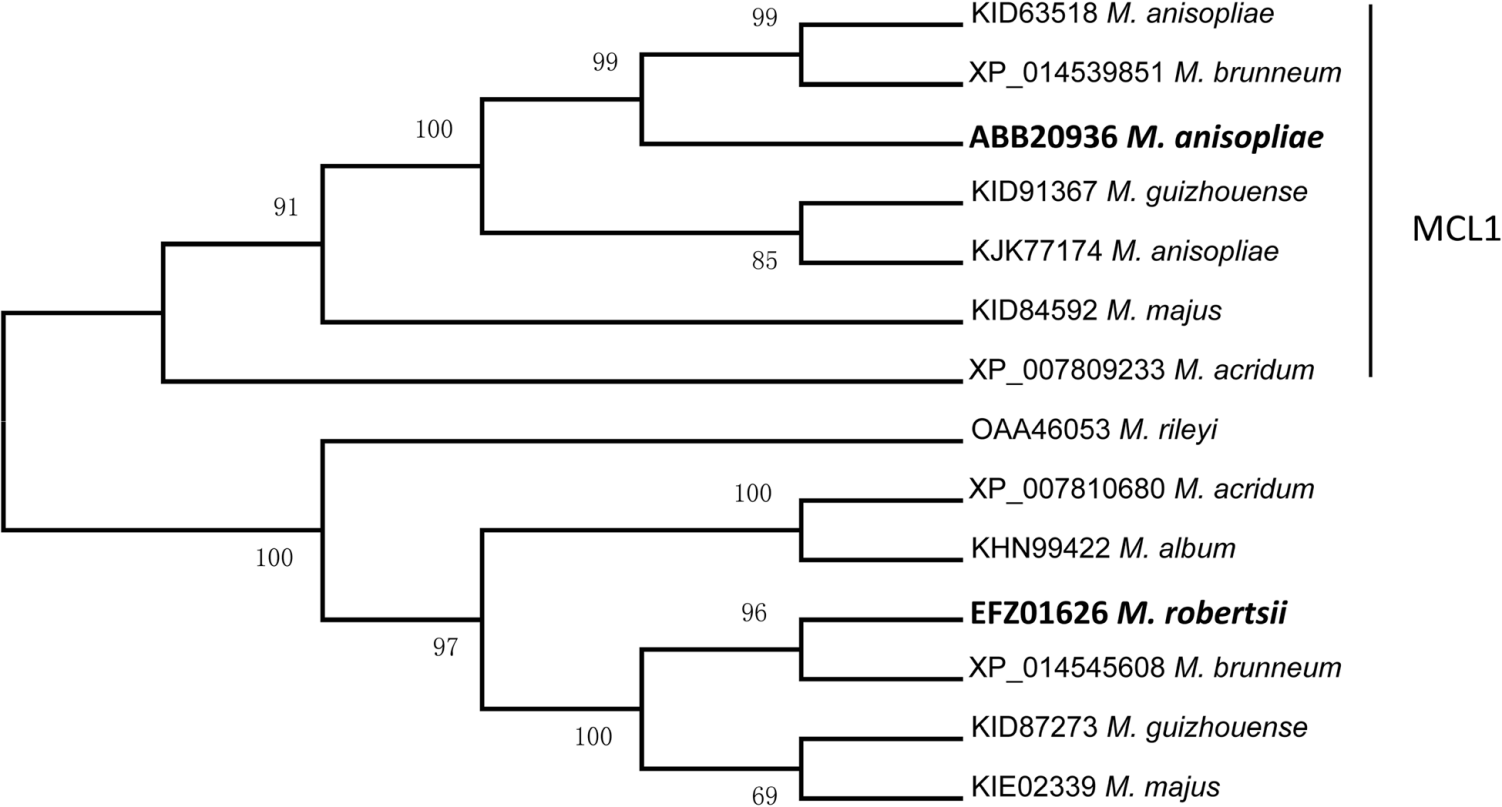
The phylogenetic tree was reconstructed by the neighbor-joining method. *M. acridum*, *Metarhizium acridum*; *M. album*, *Metarhizium album*; *M. anisopliae*, *Metarhizium anisopliae*; *M. brunneum*, *Metarhizium brunneum*; *M. guizhouense*, *Metarhizium guizhouense*; *M. majus*, *Metarhizium majus*; *M. rileyi*, *Metarhizium rileyi*; *P. chlamydosporia*, *Pochonia chlamydosporia*; *P. lilacinum*, *Purpureocillium lilacinum; T. ophioglossoides*, *Tolypocladium ophioglossoides*; *U. virens*, *Ustilaginoidea virens*

In the GenBank database, the studied gene (*Mrsvp*) was annotated as the *Mcl1* gene, which has the same name with the first reported collagen-like protein MCL1 (DQ238488) in *M. robertsii*. Phylogenetic analysis indicates that MrSVP in *M. robertsii* does not belong to the real MCL1 group (Fig. 4). Moreover, the amino acid sequence was BLASTed with the *Mcl1* gene, and homology was found to be only 29%. Next, analysis of conserved domains revealed that MrSVP is different from MCL1 in *M. anisopliae* and is not typical xMcl-1-type fungal Mcl1-related protein, which includes the characteristic Gly-X-Y repeat of the collagen domain. Most of these sequences (absent in the *D. hansenii* protein XP460045) also had a three-domain structure, including a hydrophilic 5′ domain containing variable numbers of cysteine residues, a central domain of variable numbers (22–52) of G-X-Y repeats with multiple glycosylation sites, and a 3′ domain that varies in length [10]. Accordingly, we can speculate that the *Mrsvp* gene may be incorrectly annotated as the *Mcl1* gene.

After penetration of the cuticle of an insect into the haemocoel, the entomopathogenic fungus may effectively obtain the nutrients in the host to quickly occupy the insect hemocoel. Trehalose is a nonreducing disaccharide that accounts for 80 to 90% of sugar in the hemolymph of insects and becomes the main nutrient for fungal growth after infection into hemolymph [15, 16]. Antifungal peptides, induced by a variety of bacteria and fungi, perform an important function in insect humoral immunity, which could be upregulated by fungi in the course of infection [17, 18]. In the present study, the expression of the antifungal gallerimycin (*gal*) gene from *G. mellonella* was found to be upregulated in insects infected by Δ*MrSVP*, compared with infected by WT, which is consistent with previous studies that *gal* gene was upregulated in insects infected by the fungi with gene disruption [19, 20]. In addition, the acid trehalase gene of *M. robertsii* was specifically downregulated in insect hemolymph after the disruption of *Mrsvp*. These data indicate that *Mrsvp* in fungi could facilitate the utilization of host nutrients for growth and reproduction. Therefore, we speculated the deletion of *MrSVP* resulted in the loss of the ability for the fungus to utilize the host nutrients, leaded to the host could rapid response for fungal infection, result in the high expression of the antimicrobial peptides (AMPs) such as gallerimycin.

We observed lower sporulation on insect cadavers for Δ*MrSVP* than for WT and Δ*MrSVP*/*MrSVP*; this finding is not consistent with the conidial yield of the mutant on the artificial medium. These results suggested that the ability of Δ*MrSVP* to utilize nutrients decreased in the haemocoel of insects because of downregulated acid trehalase and sucrose nonfermenting protein kinase gene (which effectively degrades trehalose in insect hemolymph) and the downregulated *snf1* gene, which regulates the derepression of glucose-repressible genes.

Fungal conidia lose viability due to exposure to high temperatures during storage, distribution, or in field applications [21]. Our previous study showed that the expression of *Mrsvp* in *M. anisopliae* increased by more than 1000-fold after heat treatment [12]. The germination rate of these spores decreased after the loss of *Mrsvp* at 42 °C heat shock treatment in this study. These data confirmed that *Mrsvp* contributes to the fungal heat response. The heat shock proteins (HSPs) are well known for imparting thermotolerance, and various HSPs have been overexpressed in *M. robertsii* to improve its tolerance of heat [22]. Of note, 10 heat tolerance–associated genes (five HSPs included) turned out to be downregulated in the Δ*MrSVP* mutant, suggesting that *Mrsvp* may serve as a critical functional factor in the fungal heat stress response via regulation of different HSPs.

Furthermore, no significant differences in the effects of oxidative, osmotic, or UV stressors or in growth and development were detected between Δ*MrSVP* and the WT *M. robertsii*, suggesting that the *Mrsvp* biosynthesis in *M. robertsii* is not involved in these pathways. We can speculate that MrSVP is a secreted virulence-associated protein rather than a participant in other pathways.

## Conclusions

Overall, our data indicate that the *Mrsvp* gene contributes to thermotolerance and virulence of *M. robertsii*. Furthermore, this gene is deeply involved in mycosis of insect cadavers and immune escape rather than insect cuticle penetration during infection. Consequently, this study should deepen the understanding of the pathogenic mechanism of action of entomopathogenic fungi and will provide a theoretical reference for improvement of the preparation quality and stability of insecticidal efficacy of *M. robertsii*.

## Methods

### Strains and media

Fungal and bacterial strains were cultured and maintained as noted before [23]. The wild-type (WT) strain, *M. robertsii* ARSEF 23, the *Agrobacterium tumefaciens* strain AGL-1, and the binary vectors pDHt-SK-bar and pDHt-SK-ben were kindly provided by Dr. Chengshu Wang [5, 24]. *M. robertsii* was routinely maintained at 25 °C on potato-dextrose-agar (PDA) to obtain conidia. For extraction of genomic DNA, cultures were grown in Sabouraud dextrose agar plus yeast extract (SDAY) at 25 °C.

### Acquisition and bioinformatics analysis of the complete gene

Sequences of the *Mrsvp* gene (EFZ01626) were obtained from the NCBI database. Based on the retrieved sequence, two pairs of gene-specific primers were designed for 5′ and 3′ rapid amplification of cDNA ends (RACE) PCR to amplify the 5′ and 3′ ends of the gene. To obtain this gene’s whole cDNA sequence, the above sequences were combined.

The molecular weight (MW) and theoretical isoelectric point (pI) were calculated using the Compute pI/Mw tool (http://www.expasy.ch/tools/pi_tool.html). The signal peptide was predicted on the SignalP 3.0 server (http://www.cbs.dtu.dk/services/SignalP/). Domain analysis was performed in the conserved domain database (https://www.ncbi.nlm.nih.gov/cdd/).

Homologous *Mrsvp* and *Mcl1* sequences from other *Metarhizium* species were retrieved from the NCBI database. For phylogenetic analysis, the deduced amino acid (aa) sequences corresponding to these sequences were analyzed by the neighbor-joining (NJ) methods in the MEGA 7 software [25].

### Construction of knockout and complemented strains

In brief, the upstream and downstream regions of *Mrsvp* were PCR-amplified from the genomic DNA of the wild type strain using the 3′ and 5′ flanking primers of the *Mrsvp* (Table 1). The *Mrsvp* deletion vector plasmid was constructed by amplifying the upstream and downstream regions, after PCR amplification, the PCR products were digested with *EcoR*I and *Pst*I as well as *Spe*I and *Xba*I, respectively, then inserted at the appropriate positions of vector pDHt-SK-bar to produce the pbar-SVP plasmid (Additional file 1: Figure S1A). Plasmids were proliferated in *E. coli* DH5α and transfected into *M. robertsii*, using *A. tumefaciens*–mediated transformation (ATMT) to knock out target genes according to previously described methods [26].

**Table 1:** PCR primers used in this study

Gene	ID^a^	5′ to 3′ sequence	Notes
Primers were designed for disruption and complementation of *Mrsvp*			
*Mrsvp*	*Mrsvp* 5′ F	CG**GAATTC**CAGACTTGCCAAATCCTAC	
	*Mrsvp* 5′ R	AA**CTGCAG**GGTGATTACCCAGTGTTCT	
	*Mrsvp* 3′ F	GG**ACTAGT**CTCTTCTGTCCAGGCCAATA	
	*Mrsvp* 3′ R	GC**TCTAGA**ACGGCTGAGGTGATGATGTA	
bar	bar F	GGAGGTCAACAATGAATGCC	PCR identification of *Mrsvp*
	bar R	CCACGTCATGCCAGTTCC	Deletion transformants
*Mrsvp*	C*Mrsvp* 5′ F (c)	GG**ACTAGT**CAGTTGGTGGCACTATTTGGT	
	C*Mrsvp* 3′ R (d)	GC**TCTAGA**TTACAAAGCGAGAACCAGAGC	
Primers were designed for the validation of mutants			
ben	ben F	ATGGCTACCTACTCCGTCGTG	PCR identification of *Mrsvp*
	ben R	CTCGTCCATACCCTCACCA	Complementation transformants
*Mrsvp*	*Mrsvp* F (a)	GATGGCAAGGGTATGGG	
	*Mrsvp* R (b)	TCAGGTTAGTAGCAGAGGGAT	

^a^Letters in brackets refer to primer positions in Additional file 1: Figure S1A; Restriction sites are in boldface

To construct the complementation vector, the PCR was carried out to amplify the *Mrsvp* gene plus 855 bp of the upstream sequences, the template was WT genomic DNA, and the primers were c and d (Additional file 1: Figure S1A). The amplicons were digested with *Spe*I and *Xba*I and inserted at the appropriate positions of pDHt-SK-ben (Additional file 1: Figure S1A). Plasmid Com-pben-SVP was mobilized in *A. tumefaciens* AGL-1 and then transfected into the mutant (loss of the *Mrsvp* gene). Table 1 lists the primers used for construction of knockout and complemented strains.

### Testing conidial tolerance to heat stress and UV stress

The proportion of fungal feasibility (conidial germination rate) under stress relative to the feasibility in the control was defined as the survival index. This procedure was performed as described in ref. [12]. Briefly, the conidial suspensions of the knockout strain, complementation strain, and WT strain were placed in a water bath for 60 min at 42 °C, followed by culturing on the PDA medium for 24 h; conidial germination was visualized under identical imaging conditions under an Olympus BX51 microscope; the conidia that had visible germ tubes were germinated.

The tolerance of UV radiation was determined by measuring the conidial germination rate following exposure to UV-B irradiation [27]. Briefly, PDA on a Petri dish (35 mm diameter) was inoculated with the conidial suspension of each strain, the PDA plates were exposed to 312 nm UV-B irradiation at gradient doses of 40–60 J/cm^− 2^. After exposure for 6 s and 48 h of incubation, germination rates were determined, and the method was alexandrine. Each treatment involved three replicates. Based on nonirradiated controls estimates, relative germination was calculated. It was computed using the counts of germinated and nongerminated conidia.

### An insect bioassay and appressorium formation

Bioassays (topical inoculation and injection) were conducted on the last instar of *G. mellonella* according to previously described methods [28]. For topical inoculation, insects were inoculated by immersion in conidial suspensions (1 × 10^7^ conidia ml^− 1^) for 60 s. Control *G. mellonella* larvae were treated with sterile distilled H_2_O. For infection, 10 μl of an aqueous suspension containing 1 × 10^6^ conidia/ml was injected into each insect. Control *G. mellonella* larvae were treated with 10 μl of sterile distilled H_2_O. All bioassays with three biological replicates, and 50 insects were used for each biological replicate.

Examination of appressorium formation was performed on a hydrophobic plastic Petri dish as described elsewhere [29] with some modifications.

### Real-time reverse-transcription PCR analysis

The expression levels of the genes related to tolerance of heat shock were analyzed by real-time reverse-transcription (RT)-PCR. Total RNA was isolated from *M. robertsii* conidia. Conidia were harvested from 14-day-old cultures on PDA plates. Tolerance rates of fungal cells to heat shock were determined as described previously, and the conidia were collected after 24 h incubation at 25 °C. Total-RNA extraction, cDNA synthesis, and real-time RT-PCR were conducted according to the previously described methods [30]. *GAPDH* (GenBank accession number EFY96862), a gene encoding glyceraldehyde 3-phosphate dehydrogenase in *M. robertsii*, served as an internal control [31]. All real-time RT-PCR reactions were carried out in triplicate for each sample, and the experiment was repeated three times.

The gallerimycin is an antifungal peptide that can be induced by a variety of bacteria, fungi. The peptide plays an important role in insect humoral immunity [20, 32]. Therefore, we examined the gallerimycin (*gal*) gene from *G. mellonella* and virulence-related genes (*pacC* [9], *mcl1* [10], *dtxS1* [33], *atm1* [34], and *snf1* [35]) of *M. robertsii* involved in mycosis of insect cadavers and evasion of host immunity. Fifteen *G. mellonella* specimens were each injected into the haemocoel with 10 μl of an aqueous suspension containing 1 × 10^8^ conidia/ml. Briefly, *G. mellonella* specimens were collected at 48 h after injection. Total-RNA extraction, cDNA synthesis, and real-time RT-PCR were conducted according to the previously described methods [19]. All experiments were repeated three times. All the primers employed for real-time RT-PCR are listed in Additional file 2: Table S1.

### Examination of the growth rate, chemical stress, and conidial yield

#### Assessments of radial growth

For the knockout strain, the complementation strain, and WT strain, aliquots of 1 μl of 1 × 10^6^ conidia ml^− 1^ suspension were centrally spotted onto the PDA, PPDA, SDAY, and 1/4 SDAY plates (9 cm diameter), maintained at 25 °C for 12 days, and then, the radial growth (colony diameter) in each plate was measured daily as described previously [19].

#### Assays for cellular responses to high osmolarity and oxidative stress

To this end, 50 μl aliquots of conidial suspensions of the WT and mutants were smeared on PDA plates, followed by 3 days of incubation at 25 °C and cutting of disks (4 mm diameter) from fungal colonies. The disks were centrally spotted onto PDA plates alone (control) or supplemented with a stressful concentration of H_2_O_2_ (2 mM) for oxidative stress, NaCl (1 M) for osmotic stress, SDS (2.5 μg ml^− 1^) and Congo red (0.3 mg ml^− 1^) [36] for cell wall stress, and carbendazim (2 μg ml^− 1^) for fungicidal stress, with culturing for 12 days at 25 °C.

#### Conidial yield

Cultures for quantification of conidiation capacity were initiated by spreading 30 μl aliquots of a 1 × 10^7^ conidia ml^− 1^ suspension on PDA (9 cm diameter) and were maintained for 14 days at 25 °C. The conidia on each plug were dislodged into 20 ml of 0.02% Tween 80 by 10 min vibration. Conidial concentration in the suspension was determined with a hemocytometer and was converted to the number of conidia per cm^2^ of plate culture as an estimate of the conidial yield.

### Statistical analysis

Relative expression levels were calculated by the comparative ΔΔCt method. The constitutively expressed *GAPDH* gene served as an internal control for each sample, and relative expression levels are shown as a ratio to those at 24 h and 48 h after inoculation respectively., which were set to 1.0 [37].

All data were analyzed in GraphPad Prism 5 (GraphPad Software, La Jolla, CA, United States). Data were expressed as the mean ± standard error (SE) of at least three independent experiments. Student’s *t* test was performed to evaluate the differences between two means. For multiple comparisons, Tukey’s multiple-comparison test was carried out for significance analysis. Data with *p* values equal to or less than 0.05 were considered significant.

## Additional files

Additional file 1: **Figure S1.** Disruption and complementation of *Mrsvp* in *M. robertsii*. (A) *Mrsvp* was disrupted by homologous recombination, and complementation of this gene was performed using a plasmid construct containing *Mrsvp*. (B) Confirmation the disruption mutants by PCR. The template is genomic DNA. *Mrsvp*, PCR was conducted with *Mrsvp* F and *Mrsvp* R; *bar*, PCR was conducted with primers bar F and bar R; *ben*, PCR was conducted with primers ben F and ben R. CK, control check; WT, wild type; Δ*MrSVP*, the knockout mutant; Comp, the complementation mutant. (C) Confirmation of the knockout mutants by PCR. The template is cDNA. Detailed information on primers is shown in Table 1. **Figure S2.** Growth and production of conidia by strains WT, Δ*MrSVP*, and Δ*MrSVP*/*MrSVP*. (A) The growth rate of WT *M. robertsii*, of the knockout mutant, and of the complementation mutant. (B) The yield of conidia was measured for the WT, Δ*MrSVP*, and Δ*MrSVP*/*MrSVP*. **Figure S3.** The effects of chemical stress reagents on growth, and the effect of UV-B irradiation on conidial viability. (A) The colony size of strains WT, Δ*MrSVP*, and of the complementation mutant in the presence of chemical stress reagents. (B) The relative percentage of germination of *M. robertsii* conidia that were exposed to UV-B radiation. Based on nonirradiated control estimates, relative germination was calculated. (ZIP 1932 kb)

Additional file 2: **Table S1.** Paired primers used for transcriptional profiling of heat shock and virulence-related genes via qPCR. (DOCX 33 kb)

## Abbreviations

MAD1: Adhesin of *Metarhizium anisopliae* to insects
MAD2: Adhesin of *Metarhizium anisopliae* enabling attachment to plants
MCL1: A protein similar to host collagen secreted by *Metarhizium anisopliae*
MOS1: *Metarhizium anisopliae* osmosensor protein
MrSVP: *Metarhizium robertsii* secreted virulence-associated protein
PPDA: Potato peptone dextrose agar medium.
RACE: Rapid amplification of cDNA ends

## References

[1] Roberts DW, St Leger RJ (2004). Metarhizium spp., cosmopolitan insect pathogenic fungi: mycological aspects. Adv Appl Microbiol.

[2] Chen X, Xu C, Qian Y, Liu R, Zhang Q, Zeng G, Zhang X, Zhao H, Fang W (2016). MAPK cascade-mediated regulation of pathogenicity, conidiation and tolerance to abiotic stresses in the entomopathogenic fungus Metarhizium robertsii. Environ Microbiol.

[3] MRd F, Wraight SP (2007). Mycoinsecticides and Mycoacaricides: a comprehensive list with worldwide coverage and international classification of formulation types. Biol Control.

[4] Xiao H, Guohua X, Peng Z, Yanfang S, Yao S, Xinyu Z, Xingzhong L, Zhan S, Leger RJS, Wang C (2014). Trajectory and genomic determinants of fungal-pathogen speciation and host adaptation. Proc Natl Acad Sci U S A.

[5] Gao QA, Jin K, Ying SH, Zhang YJ, Xiao GH, Shang YF, Duan ZB, Hu XA, Xie XQ, Zhou G (2011). Genome sequencing and comparative transcriptomics of the model Entomopathogenic Fungi Metarhizium anisopliae and M. acridum. Plos Genet.

[6] Charnley AK, St Leger RJ (1991). The role of cuticle-degrading enzymes in fungal pathogenesis in insects. Cole E T, Hoch H C. Fungal Spore Disease Initiation in Plants and Animals.

[7] Fang W, Bidochka MJ (2006). Expression of genes involved in germination, conidiogenesis and pathogenesis in Metarhizium anisopliae using quantitative real-time RT-PCR. Mycol Res.

[8] Wang C, St Leger RJ (2007). The MAD1 adhesin of Metarhizium anisopliae links adhesion with blastospore production and virulence to insects, and the MAD2 adhesin enables attachment to plants. Eukaryot Cell.

[9] Huang W, Shang Y, Chen P, Gao Q, Wang C (2015). MrpacC regulates sporulation, insect cuticle penetration and immune evasion in Metarhizium robertsii. Environ Microbiol.

[10] Wang C, St Leger RJ (2006). A collagenous protective coat enables Metarhizium anisopliae to evade insect immune responses. Proc Natl Acad Sci U S A.

[11] Wang C, Duan Z, St Leger RJ (2008). MOS1 osmosensor of Metarhizium anisopliae is required for adaptation to insect host hemolymph. Eukaryot Cell.

[12] Wang Z, Zhou X, Meng H (2014). Comparative transcriptomic analysis of the heat stress response in the filamentous fungus Metarhizium anisopliae using RNA-Seq. Appl Microbiol Biotechnol.

[13] Rustiguel CB, Rosa JC, Jorge JA, de Oliveira AH, Guimaraes LH (2016). Secretome analysis of Metarhizium anisopliae under submerged conditions using Bombyx mori Chrysalis to induce expression of virulence-related proteins. Curr Microbiol.

[14] Wang JJ, Cai Q, Qiu L, Ying SH, Feng MG (2018). The histone acetyltransferase Mst2 sustains the biological control potential of a fungal insect pathogen through transcriptional regulation. Appl Microbiol Biotechnol.

[15] Xia Y, Clarkson JM, Charnley AK (2002). Trehalose-hydrolysing enzymes of Metarhizium anisopliae and their role in pathogenesis of the tobacco hornworm, Manduca Sexta. J Invertebr Pathol.

[16] Zhao H, Charnley AK, Wang Z, Yin Y, Li Z, Li Y, Cao Y, Peng G, Xia Y (2006). Identification of an extracellular acid trehalase and its gene involved in fungal pathogenesis of Metarizium anisopliae. J Biochem.

[17] Wang C, Wang S (2017). Insect pathogenic Fungi: genomics, molecular interactions, and genetic improvements. Annu Rev Entomol.

[18] Dubovskiy IM, Whitten MM, Yaroslavtseva ON, Greig C, Kryukov VY, Grizanova EV, Mukherjee K, Vilcinskas A, Glupov VV, Butt TM (2013). Can insects develop resistance to insect pathogenic fungi?. PLoS One.

[19] Zhou R, Zhou X, Fan A, Wang Z, Huang B (2018). Differential functions of two metalloproteases, Mrmep1 and Mrmep2, in growth, sporulation, Cell Wall integrity, and virulence in the filamentous fungus Metarhizium robertsii. Front Microbiol.

[20] Cen K, Li B, Lu Y, Zhang S, Wang C (2017). Divergent LysM effectors contribute to the virulence of Beauveria bassiana by evasion of insect immune defenses. PLoS Pathog.

[21] Rangel DE, Alston DG, Roberts DW (2008). Effects of physical and nutritional stress conditions during mycelial growth on conidial germination speed, adhesion to host cuticle, and virulence of Metarhizium anisopliae, an entomopathogenic fungus. Mycol Res.

[22] Liao X, Lu HL, Fang W, St Leger RJ (2014). Overexpression of a Metarhizium robertsii HSP25 gene increases thermotolerance and survival in soil. Appl Microbiol Biotechnol.

[23] Wang Y, Wang T, Qiao L, Zhu J, Fan J, Zhang T, Wang ZX, Li W, Chen A, Huang B (2017). DNA methyltransferases contribute to the fungal development, stress tolerance and virulence of the entomopathogenic fungus Metarhizium robertsii. Appl Microbiol Biotechnol.

[24] Gao Q, Shang YF, Huang W, Wang CS (2013). Glycerol-3-phosphate acyltransferase contributes to triacylglycerol biosynthesis, lipid droplet formation, and host invasion in Metarhizium robertsii. Appl Environ Microbiol.

[25] Kumar S, Stecher G, Tamura K (2016). MEGA7: molecular evolutionary genetics analysis version 7.0 for bigger datasets. Mol Biol Evol.

[26] Duan Z, Chen Y, Huang W, Shang Y, Chen P, Wang C (2013). Linkage of autophagy to fungal development, lipid storage and virulence in Metarhizium robertsii. Autophagy.

[27] Yao S-L, Ying S-H, Feng M-G, Hatting JL (2010). In vitro and in vivo responses of fungal biocontrol agents to gradient doses of UV-B and UV-A irradiation. BioControl.

[28] Fang W, Fernandes EK, Roberts DW, Bidochka MJ, St Leger RJ (2010). A laccase exclusively expressed by Metarhizium anisopliae during isotropic growth is involved in pigmentation, tolerance to abiotic stresses and virulence. Fungal Genet Biol.

[29] He M, Xia Y (2009). Construction and analysis of a normalized cDNA library from Metarhizium anisopliae var. acridum germinating and differentiating on Locusta migratoria wings. FEMS Microbiol Lett.

[30] Meng H, Wang Z, Wang Y, Zhu H, Huang B (2017). Dicer and Argonaute genes involved in RNA interference in the Entomopathogenic fungus Metarhizium robertsii. Appl Environ Microbiol.

[31] Fang W, Pei Y, Bidochka MJ (2006). Transformation of Metarhizium anisopliae mediated by agrobacterium tumefaciens. Can J Microbiol.

[32] Feng P, Shang Y, Cen K, Wang C (2015). Fungal biosynthesis of the bibenzoquinone oosporein to evade insect immunity. Proc Natl Acad Sci U S A.

[33] Wang B, Kang Q, Lu Y, Bai L, Wang C (2012). Unveiling the biosynthetic puzzle of destruxins in Metarhizium species. Proc Natl Acad Sci U S A.

[34] Jin K, Peng G, Liu Y, Xia Y (2015). The acid trehalase, ATM1, contributes to the in vivo growth and virulence of the entomopathogenic fungus, Metarhizium acridum. Fungal Genet Biol.

[35] Ming Y, Wei Q, Jin K, Xia Y (2014). MaSnf1, a sucrose non-fermenting protein kinase gene, is involved in carbon source utilization, stress tolerance, and virulence in Metarhizium acridum. Appl Microbiol Biotechnol.

[36] Ying SH, Feng MG (2011). A conidial protein (CP15) of Beauveria bassiana contributes to the conidial tolerance of the entomopathogenic fungus to thermal and oxidative stresses. Appl Microbiol Biotechnol.

[37] Livak KJ, Schmittgen TD (2001). Analysis of relative gene expression data using real-time quantitative PCR and the 2(−Delta Delta C(T)) method. Methods.

