# Supplementary material for: MrSVP, a secreted virulence-associated protein, contributes to thermotolerance and virulence of the entomopathogenic fungus Metarhizium robertsii
Source: BMC Microbiol. 2019 Jan 28;19:25. doi: 10.1186/s12866-019-1396-8 (PMC6350332; doi:10.1186/s12866-019-1396-8)
Supplement: Supplementary file 1 — Figure S1. Disruption and complementation of Mrsvp in M. robertsii. (A) Mrsvp was disrupted by homologous recombination, and complementation of this gene was performed using a plasmid construct containing Mrsvp. (B) Confirmation the disruption mutants by PCR. The template is genomic DNA. Mrsvp, PCR was conducted with Mrsvp F and Mrsvp R; bar, PCR was conducted with primers bar F and bar R; ben, PCR was conducted with primers ben F and ben R. CK, control check; WT, wild type; ΔMrSVP, the knockout mutant; Comp, the complementation mutant. (C) Confirmation of the knockout mutants by PCR. The template is cDNA. Detailed information on primers is shown in Table 1. Figure S2. Growth and production of conidia by strains WT, ΔMrSVP, and ΔMrSVP/MrSVP. (A) The growth rate of WT M. robertsii, of the knockout mutant, and of the complementation mutant. (B) The yield of conidia was measured for the WT, ΔMrSVP, and ΔMrSVP/MrSVP. Figure S3. The effects of chemical stress reagents on growth, and the effect of UV-B irradiation on conidial viability. (A) The colony size of strains WT, ΔMrSVP, and of the complementation mutant in the presence of chemical stress reagents. (B) The relative percentage of germination of M. robertsii conidia that were exposed to UV-B radiation. Based on nonirradiated control estimates, relative germination was calculated. (ZIP 1932 kb) [file 12866_2019_1396_MOESM1_ESM.zip › Supplementary material figure legend .docx]

**Supplementary material figure legend**

**Figure S1.** Disruption and complementation of *Mrsvp* in *M. robertsii*. (A) *Mrsvp* was disrupted by homologous recombination, and complementation of this gene was performed using a plasmid construct containing *Mrsvp*. (B) Confirmation the disruption mutants by PCR. The template is genomic DNA. *Mrsvp*, PCR was conducted with *Mrsvp* F and *Mrsvp* R; *bar*, PCR was conducted with primers bar F and bar R; *ben*, PCR was conducted with primers ben F and ben R. CK, control check; WT, wild type; Δ*MrSVP*, the knockout mutant; Comp, the complementation mutant. (C) Confirmation of the knockout mutants by PCR. The template is cDNA. Detailed information on primers is shown in Table 1.

**Figure S2.** Growth and production of conidia by strains WT, Δ*MrSVP*, and Δ*MrSVP*/*MrSVP*. (A) The growth rate of WT *M. robertsii*, of the knockout mutant, and of the complementation mutant. (B) The yield of conidia was measured for the WT, Δ*MrSVP*, and Δ*MrSVP*/*MrSVP*.

**Figure S3.** The effects of chemical stress reagents on growth, and the effect of UV-B irradiation on conidial viability. (A) The colony size of strains WT, Δ*MrSVP*, and of the complementation mutant in the presence of chemical stress reagents. (B) The relative percentage of germination of *M. robertsii* conidia that were exposed to UV-B radiation. Based on nonirradiated control estimates, relative germination was calculated.
