# Supplementary material for: MrSVP, a secreted virulence-associated protein, contributes to thermotolerance and virulence of the entomopathogenic fungus Metarhizium robertsii
Source: BMC Microbiol. 2019 Jan 28;19:25. doi: 10.1186/s12866-019-1396-8 (PMC6350332; doi:10.1186/s12866-019-1396-8)
Supplement: Supplementary file 2 — Table S1. Paired primers used for transcriptional profiling of heat shock and virulence-related genes via qPCR. (DOCX 33 kb) [file 12866_2019_1396_MOESM2_ESM.docx]

**Supplementary material table 1. Paired primers used for transcriptional profiling of heat shock and virulence-related genes via qPCR.**

| **Gene** | | **Accession number** | | **Annotation** | **Sequence of primer sets (5′–3′)** | |  |
| --- | --- | --- | --- | --- | --- | --- | --- |
| **Involved in virulence in fungus** | | | | | | |  |
| *pacC* | | MAA_06038 | | PacC transcription factor | GTTAGTGAAACCACCAGA / TTGACGAAATCCTTGC | |  |
| *mcl1* | | MAA_01665 | | Collagenous | CTCCAAGCCAACTCGC / GAGGAAGAGCATAGCCAC | |  |
| *dtxS1* | | MAA_10043 | | destruxin synthetase | GGACCAAGGTTCTGACA / TGCGGCAAATCTATCTC | |  |
| *atm1* | | MAA_09549 | | acid trehalase | ACGCTGCTGATTACCGG / CCATCCATCTTCCTCCTCT | |  |
| *snf1* | | MAA_04401 | | sucrose non-fermenting protein kinase gene | ACGAGTACGACGGCACA / TCCCACCGCACTTTGA | |  |
| *gal* | | AF453824 | | Gallerimycin | CTACAGAATCACACGACACT / CGAAGACATTGACATCCATT | |  |
| **Response to heat shock** | | | | | | | |
| *hsp30a* | MAA_07190 | | Heat shock protein 20 | | | CTTCGCACAAGGCCACC / GAGAACTCGCCGACGCT | |
| *hsp30b* | MAA_04014 | | Heat shock protein 30 | | | CTGAGCCCGAGGAGAAG / GACACGGTTGGGAAAGT | |
| *hsp60* | MAA_07685 | | Heat shock protein 60 | | | CGGCCAACTTTGACCAG / CAATGACAACGGAACCCT | |
| *hsp90* | MAA_04726 | | Heat shock protein 90 | | | TATGTCCGCCGTGTCTT / TGTTCTGCTGGAGGGTC | |
| *hsp104* | MAA_03534 | | Heat shock protein 104 | | | TCTGCGGTCCCTCTGGT / CGGCTAAGTGCGTGTCG | |
| *tps1* | MAA_04676 | | glycosyltransferase family 20 protein | | | GTATGCCTCGTCTCCTCG / GCCAGTTCCTCCGTGTT | |
| *tps2* | MAA_07933 | | glycosyltransferase family 20 protein | | | CAACAAACTCGGCAAAC / ACTTAGGACACCGCTCAC | |
| *nth2* | MAA_03359 | | glycoside hydrolase family 37 protein | | | GAGAACGGCTGGGACT / CAGGATGGAGTTGAGGC | |
| *mtd* | MAA_09034 | | L-xylulose reductase | | | GTCATCACGGCGTCCAT / GAGGCCAGTGTCGATGTAG | |
| *mpd* | MAA_08216 | | mannitol-1-phosphate dehydrogenase | | | ACGAGTTTGAGGAGGATG / CGAGAAAGTTTACGCAGAG | |
